# When the Apex Deceives: A Mobile Left Ventricular Mass After Myocardial Infarction

**DOI:** 10.3390/reports9020124

**Published:** 2026-04-18

**Authors:** Georgios E. Zakynthinos, George Makavos, Nikolaos K. Kokkinos, Ourania Katsarou, Evangelos Oikonomou, Gerasimos Siasos

**Affiliations:** 3rd Department of Cardiology, Thoracic Diseases Hospital of Athens “Sotiria”, National and Kapodistrian University of Athens, 11527 Athens, Greece; gmakavos@hotmail.com (G.M.); nikoskokkinoss3@hotmail.gr (N.K.K.); raniakatsarou@yahoo.gr (O.K.); boikono@gmail.com (E.O.); gersiasos@gmail.com (G.S.)

**Keywords:** left ventricular thrombus, contrast echocardiography, myocardial infarction, mechanical complications, acute heart failure, intracardiac mass, anticoagulation, diagnostic imaging

## Abstract

**Background and Clinical Significance:** Mechanical complications and intracavitary thrombus are both recognized causes of clinical deterioration following acute myocardial infarction, yet they require fundamentally different therapeutic approaches. Distinguishing between these entities is critical, as misdiagnosis may lead to unnecessary surgical intervention or delayed anticoagulation with serious consequences. Left ventricular (LV) thrombus typically appears as a well-defined mass; however, atypical and highly mobile morphologies may closely mimic catastrophic post-infarction mechanical complications, creating significant diagnostic uncertainty. This case highlights the pivotal role of contrast-enhanced echocardiography in resolving such ambiguity and guiding appropriate management in a high-stakes clinical setting. **Case Presentation:** A 60-year-old man presented with acute dyspnea and pulmonary edema ten days after an anterior myocardial infarction treated with percutaneous coronary intervention, complicated by ischemic stroke. Transthoracic echocardiography demonstrated severe LV systolic dysfunction with moderate-to-severe mitral regurgitation and an unexpected, highly mobile, irregular mass protruding into the LV apex. The mass exhibited a shredded, tissue-like appearance, raising urgent concern for post-infarction mechanical complications, including papillary muscle rupture or apical myocardial disruption, and prompting immediate consideration of surgical intervention. Contrast-enhanced echocardiography was performed and revealed a mobile LV apical thrombus. Surgical management was avoided, and systemic anticoagulation was initiated, followed by transition to rivaroxaban in combination with ongoing dual antiplatelet therapy. The patient demonstrated rapid clinical improvement with optimized heart failure treatment and was discharged after four days, with planned follow-up imaging to assess thrombus resolution. **Conclusions:** Left ventricular thrombus may present with atypical, misleading morphologies that closely resemble life-threatening mechanical complications after myocardial infarction.

## 1. Introduction and Clinical Significance

Left ventricular thrombus (LVT) is a well-recognized complication following extensive myocardial infarction, particularly in the presence of severe systolic dysfunction and regional wall motion abnormalities [1]. In the contemporary primary percutaneous coronary intervention (PCI) era, the reported incidence of LVT ranges from approximately 2–10%, although higher rates are observed in patients with extensive anterior infarction and markedly reduced left ventricular ejection fraction [1,2].

While its classical appearance is often mural and laminated, LVT can occasionally adopt unusual, highly mobile morphologies that mimic post-infarction mechanical complications such as papillary muscle rupture or apical myocardial dissection [1,2]. Distinguishing between these entities is critical, as they carry dramatically different management implications—urgent surgery versus anticoagulation [2].

We report the case of a 60-year-old man with acute pulmonary edema and a highly mobile, irregular apical mass following anterior myocardial infarction and percutaneous coronary intervention, demonstrating an unusual thrombus morphology that closely mimicked a mechanical complication and highlighting the pivotal role of contrast echocardiography in guiding appropriate treatment.

## 2. Case Presentation

A 60-year-old man presented to the emergency department with sudden-onset dyspnea and progressive respiratory distress. Earlier that morning, he had experienced a brief episode of left-sided numbness and weakness, raising concern for a transient ischemic attack. His history included coronary artery disease and a recent myocardial infarction treated with PCI to the left anterior descending (LAD) artery 10 days earlier, complicated by an ischemic stroke during the same hospitalization. He remained on dual antiplatelet therapy and was also receiving acenocoumarin, although the indication was unclear. The prior hospitalization had occurred at another institution and complete records were not available; it was hypothesized that anticoagulation may have been initiated for suspected apical thrombus in the context of the ischemic stroke, although this could not be confirmed.

At presentation, he was afebrile and hemodynamically stable (BP 118/74 mmHg, HR 100 bpm), though notably tachypneic at 30/min with SpO_2_ 94% on room air. His examination revealed acute decompensated heart failure and pulmonary edema, with elevated JVP and diffuse lung crackles. ECG demonstrated sinus tachycardia without acute ischemic changes. Laboratory evaluation showed a subtherapeutic INR of 1.8; the patient reported that INR levels had not been reassessed following discharge from the prior hospitalization. Apart from an elevated BNP, no other significant laboratory abnormalities were identified.

He responded well to intravenous loop diuretics, but his clinical presentation prompted urgent imaging.

Transthoracic echocardiography demonstrated a markedly dilated LV with severely reduced systolic function (EF 30%) and LV end-diastolic diameter of 66 mm. Regional wall motion abnormalities extended across the LAD and inferior territories, while basal and mid posterolateral segments appeared preserved, and moderate to severe mitral regurgitation was present. The regurgitant jet was central without clear echocardiographic evidence of papillary muscle rupture or primary mitral valve pathology, suggesting secondary mitral regurgitation. However, an additional significant finding was identified.

**Figure 1:**
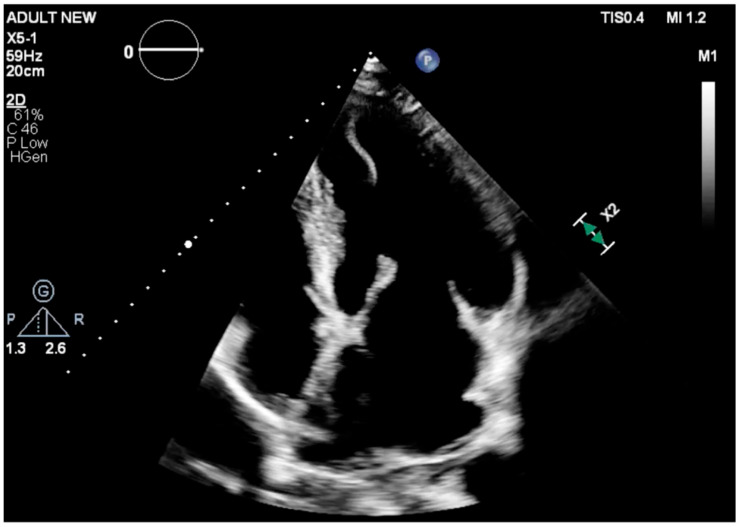
Apical four-chamber transthoracic echocardiographic view revealing a mobile, irregular mass attached to the left ventricular apex.

Within the LV apex, a large, highly mobile intracavitary mass with irregular morphology was present, with irregular, filamentous morphology projected into the cavity (Figure 1; Videos S1 and S2). The structure measured approximately 30 mm in length. With each cardiac cycle, it moved freely—appearing nearly detached from the surrounding myocardium—raising immediate concern for:

• Ruptured papillary muscle fragment;
• Apical myocardial dissection;
• Necrotic, delaminated trabeculation;
• Or a large, unstable LV thrombus following myocardial infarction.

The morphology was ambiguous enough that cardiac surgeons were pre-alerted while the cardiology team debated the differential.

## 3. Diagnosis

A large, protruding, and highly mobile left ventricular apical thrombus was identified.

Given the high stakes—anticoagulation versus emergent surgery—contrast-enhanced echocardiography was urgently performed.

Contrast-enhanced echocardiography using sulfur hexafluoride microbubble contrast (SonoVue^®^Bracco Imaging S.p.A., Milan, Italy) was therefore performed according to the standard protocol for suspected left ventricular thrombus, using low–mechanical index imaging to assess contrast uptake within the mass.

Contrast vividly opacified the LV cavity, yet the apical structure remained a sharply demarcated filling defect with no intralesional contrast uptake, excluding myocardial rupture, apical dissection, or necrotic muscle (Videos S3 & S4). What initially appeared to represent a fragment of disrupted myocardium was clearly demonstrated to be a high-risk, mobile apical thrombus.

At the outset of the diagnostic workup, cardiac magnetic resonance imaging (MRI) had been considered as a complementary modality for tissue characterization and differentiation between thrombus and mechanical complications. Although MRI is considered a reference modality for thrombus characterization, the diagnostic uncertainty was rapidly resolved with contrast echocardiography, which provided immediate bedside clarification in the acute clinical setting. Given the patient’s presentation with acute heart failure and the need for urgent decision-making regarding possible surgical intervention, additional imaging with MRI was not pursued.

This clarification of the diagnosis promptly redirected management toward systemic anticoagulation and prevented unnecessary and potentially harmful surgical intervention.

Therapeutic anticoagulation was initiated with low-molecular-weight heparin and subsequently transitioned to rivaroxaban (20 mg) in combination with dual antiplatelet therapy (acetylsalicylic acid 80 mg and clopidogrel 75 mg), considering the subtherapeutic INR on acenocoumarin and concerns regarding reliable anticoagulation monitoring.

Review of his most recent coronary angiography revealed three-vessel disease, including a chronic total occlusion of the right coronary artery, while the previously placed LAD and LCx stents remained patent. Given the recent PCI and the findings on coronary angiography, the patient was considered to have a high thrombotic risk and low bleeding risk. A strategy of prolonged short-term triple therapy was therefore adopted, with anticoagulation and dual antiplatelet therapy continued for one month, followed by discontinuation of the second antiplatelet agent thereafter. The patient showed steady clinical improvement with optimization of heart failure management, including reduction in the severity of mitral regurgitation following diuretic therapy and volume status correction, consistent with functional MR secondary to elevated LV end-diastolic pressure and acute left ventricular dilation, and was discharged after four days.

Neurological evaluation was also performed. A head CT showed no evidence of new acute ischemic lesions, demonstrating only findings consistent with a chronic–subacute infarct likely related to the previously documented stroke. Given the transient neurological symptoms at presentation, repeat CT imaging was performed after 48 h and showed no interval changes. A brain MRI was scheduled as part of the outpatient follow-up evaluation.

Follow-up echocardiography was planned according to ESC guideline recommendations for left ventricular thrombus, with repeat imaging at approximately 3 months to assess thrombus resolution, while an earlier clinical reassessment at 2 weeks was performed to evaluate medical therapy and heart failure status, without repeat echocardiographic imaging at that time.

## 4. Discussion

Left ventricular thrombus typically develops in the context of severe regional wall motion abnormalities, extensive myocardial infarction (MI), or markedly impaired systolic function. These structural and hemodynamic disturbances promote blood stasis and thrombogenesis, increasing the risk of systemic embolization [1]. Although the appearance of LVT is often characteristic, thrombi can occasionally adopt unusual morphologies that complicate differentiation from post-infarction mechanical complications [2].

In the present case, several clinical features raised concern for papillary muscle rupture or apical myocardial disruption. The patient presented with acute pulmonary edema and new mitral regurgitation (MR), findings commonly associated with papillary muscle rupture. Importantly, the myocardial infarction had occurred only 10 days earlier, a typical time frame for papillary muscle necrosis and structural failure [3]. Against this clinical background, the echocardiographic finding of a highly mobile apical mass raised immediate concern for a mechanical complication.

The mass demonstrated an irregular, filamentous morphology and moved almost independently from the surrounding myocardium. This appearance closely resembled a ruptured papillary muscle fragment or myocardial tissue disruption. Such a configuration differs from the typical presentation of LVT, which most commonly appears as a mural or laminated mass adherent to infarcted myocardium [2].

Transthoracic echocardiography is the primary imaging modality for suspected LVT; however, in cases with distorted ventricular anatomy or atypical morphology, diagnostic uncertainty may arise [2]. In this case, the combination of recent MI, flash pulmonary edema, new MR, and a pendulous intracavitary mass created a clinical picture strongly suggestive of structural myocardial failure rather than thrombus.

Contrast-enhanced echocardiography proved to be the diagnostic turning point. The technique clearly demonstrated the avascular nature of the mass, confirming the diagnosis of LVT and excluding myocardial rupture or apical dissection. This finding immediately redirected management toward systemic anticoagulation and avoided emergent surgical intervention.

This clarification had immediate therapeutic implications. Prior to contrast imaging, the clinical presentation and echocardiographic appearance raised substantial concern for a mechanical complication of myocardial infarction, particularly papillary muscle rupture or myocardial structural disruption. Such conditions typically require urgent surgical management and therefore prompted early surgical awareness while the diagnostic evaluation was ongoing. Once contrast echocardiography demonstrated that the mass represented an avascular thrombus rather than myocardial tissue, the possibility of mechanical rupture was effectively excluded. As a result, management shifted from consideration of emergent surgical intervention to systemic anticoagulation for high-risk left ventricular thrombus.

Another important element in this case is the anticoagulation history. The patient had been receiving acenocoumarin, a vitamin K antagonist traditionally considered first-line therapy for LV thrombus. Nevertheless, he presented with a large, highly mobile thrombus, suggesting either subtherapeutic anticoagulation or an exceptionally thrombogenic ventricular environment [4]. This apparent “failure” of VKA therapy supported the decision to transition to a direct oral anticoagulant (DOAC), specifically rivaroxaban.

Growing evidence supports the use of DOACs for the treatment of LVT [5,6]. Several observational studies and recent meta-analyses have demonstrated comparable or improved thrombus resolution rates and a reduced bleeding risk compared with warfarin [6]. Rivaroxaban has shown favorable outcomes in pooled analyses, offering a more stable therapeutic effect without the need for frequent monitoring [5]. Current expert consensus therefore considers DOACs a reasonable alternative in patients with unstable INR control or difficulty maintaining therapeutic anticoagulation.

Recent advances in artificial intelligence (AI) may further enhance diagnostic precision in complex cardiovascular imaging scenarios. AI-driven algorithms applied to echocardiography and cardiac imaging datasets have demonstrated potential for differentiating intracardiac masses, characterizing thrombus morphology, and supporting therapeutic decision-making through large-scale pattern recognition and risk stratification [7]. Such tools may be particularly valuable in atypical presentations like the present case.

Follow-up imaging, typically echocardiography, is required to confirm thrombus resolution and guide the duration of anticoagulation therapy. In the present case, repeat imaging was scheduled at three months according to guideline recommendations.

This case highlights an important diagnostic pitfall: left ventricular thrombus can closely mimic the appearance and clinical presentation of papillary muscle rupture following recent myocardial infarction. Awareness of this overlap is essential, as correct diagnosis can prevent unnecessary surgery and ensure timely initiation of appropriate anticoagulation therapy.

## 5. Conclusions

Contrast-enhanced echocardiography is a valuable tool in the evaluation of ambiguous intracavitary masses following myocardial infarction. Left ventricular thrombus may occasionally present with atypical morphology that mimics mechanical complications such as papillary muscle rupture or apical dissection. In such situations, contrast echocardiography can provide rapid diagnostic clarification, allowing appropriate anticoagulation therapy while avoiding unnecessary surgical intervention.

## Data Availability

The original data presented in this study are available on reasonable request from the corresponding author. The data are not publicly available due to privacy concerns.

## Supplementary Materials

The following supporting information can be downloaded at: https://www.mdpi.com/article/10.3390/reports9020124/s1, Video S1: Standard apical four-chamber transthoracic echocardiography demonstrating a mobile irregular mass at the LV apex. Video S2: Apical two-chamber transthoracic echocardiographic view in which the left ventricular apical mass is only partially visualized due to limited image clarity. Video S3 & S4: Contrast-enhanced echocardiography image demonstrating intracavitary filling defect consistent with LV thrombus.

## Abbreviations

The following abbreviations are used in this manuscript:

ACS	Acute Coronary Syndrome
DOAC	Direct Oral Anticoagulant
ECG	Electrocardiogram
EF	Ejection Fraction
ICU	Intensive Care Unit
INR	International Normalized Ratio
LAD	Left Anterior Descending artery
LV	Left Ventricle/Left Ventricular
LVT	Left Ventricular Thrombus
MI	Myocardial Infarction
MR	Mitral Regurgitation
PCI	Percutaneous Coronary Intervention
TTE	Transthoracic Echocardiography
VKA	Vitamin K Antagonist

## References

[1] McCarthy C.P., Murphy S., Venkateswaran R.V., Singh A., Chang L.L., Joice M.G., Rivero J.M., Vaduganathan M., Januzzi J.L., Bhatt D.L. (2019). Left Ventricular Thrombus: Contemporary Etiologies, Treatment Strategies, and Outcomes. J. Am. Coll. Cardiol..

[2] Srichai M.B., Junor C., Rodriguez L.L., Stillman A.E., Grimm R.A., Lieber M.L., Weaver J.A., Smedira N.G., White R.D. (2006). Clinical, imaging, and pathological characteristics of left ventricular thrombus: A comparison of contrast-enhanced magnetic resonance imaging, transthoracic echocardiography, and transesophageal echocardiography with surgical or pathological validation. Am. Heart J..

[3] Gianstefani S., Douiri A., Delithanasis I., Rogers T., Sen A., Kalra S., Charangwa L., Reiken J., Monaghan M., MacCarthy P. (2014). Incidence and predictors of early left ventricular thrombus after ST-elevation myocardial infarction in the contemporary era of primary percutaneous coronary intervention. Am. J. Cardiol..

[4] Di Odoardo L.A.F., Bianco M., Gil I.J.N., Motolese I.G., Chinaglia A., Vicenzi M., Carugo S., Stefanini G.G., Cerrato E. (2024). Left Ventricular Thrombus Management After Acute Myocardial Infarction in Clinical Practice: Results from LEVITATION Survey and Narrative Review. Cardiovasc. Drugs Ther..

[5] Magdy J., He M., Arockiam S., Harada N., Wheatcroft S.B., Bulluck H. (2025). An Updated Meta-Analysis of Randomized Controlled Trials Comparing Direct Oral Anticoagulants Against Warfarin for Left Ventricular Thrombus Resolution. J. Clin. Med..

[6] Salah H.M., Goel A., Saluja P., Voruganti D., Al’Aref S.J., Paydak H., Devabhaktuni S.R. (2021). Direct Oral Anticoagulants Versus Warfarin in Left Ventricular Thrombus: A Systematic Review and Meta-Analysis. Am. J. Ther..

[7] Aşkın L., Polat E., Hoşoğlu Y., Tanrıverdi O. (2024). The application of artificial intelligence in the field of cardiovascular diseases focuses on both diagnostic and therapeutic aspects. Exp. Appl. Med. Sci..

